# PATELLAR FRACTURE IN ANTERIOR CRUCIATE LIGAMENT RECONSTRUCTION: IN VITRO ANALYSIS

**DOI:** 10.1590/1413-785220233102e259557

**Published:** 2023-05-01

**Authors:** MARCELO DE ALMEIDA FERRER, MARIANA DE OLIVEIRA LOBO, LAÍS MARIA PINTO ALMEIDA, ANDERSON FREITAS, SÍLVIO LEITE DE MACEDO NETO, LEONARDO MORAIS PAIVA, LEONARDO RIGOBELLO BATTAGLION

**Affiliations:** 1 Clínica OrtoSul, Brasília, DF, Brazil.; 2 Hospital Regional do Gama, Serviço de Residência Médica em Ortopedia e Traumatologia, Brasília, DF, Brazil.; 3 Centro Universitário Tiradentes, Maceió, AL, Brazil.; 4 Hospital Regional do Gama, Brasília, DF, Brazil.; 5 Instituto de Pesquisa e Ensino HOME, Brasília, DF, Brazil.; 6 Universidade de Sao Paulo, Faculdade de Medicina de Ribeirão Preto, Ribeirão Preto, SP, Brazil.

**Keywords:** Biomechanical Phenomena, Finite Element Analysis, Bone-Patellar Tendon-Bone Grafting, Anterior Cruciate Ligament Injuries, Fenômenos Biomecânicos, Análise de Elementos Finitos, Enxerto Osso-Tendão Patelar-Osso, Lesões do Ligamento Cruzado Anterior

## Abstract

**Objective:**

To determine, by biomechanical analysis, safe patellar cut limits in anterior cruciate ligament (ACL) reconstruction that minimize fracture risks.

**Methods:**

From three-dimensional reconstruction, triangular cuts were made in the patella, with a depth of 6.5 mm and variable width and length (10 to 20 mm and 8 to 12 mm, respectively, both with an interval of 1 mm). The combinations of cuts constituted 55 models for tests, with five variations in width and 11 variations in length, tested with the finite element method (FEM).

**Results:**

The mean of the localized principal maximum (traction force) values was 4.36 Pa (SD 0.87 ± 0.76) and the localized principal minimum (compression force) was −4.33 Pa (SD 1.05 ± 1.11). Comparing width and length to the tension force of the values of the main maximum, we found statistical significance from 11 mm for width and 13 mm for length.

**Conclusion:**

In ACL reconstruction, the removal of the patellar bone fragment is safe for fragments smaller than 11 mm in width and 13 mm in length, which corresponds to 24% of the width and 28% of the length of the patella used. ***Level of Evidence II, Comparative Prospective Study.***

## RESUMO

**Objetivo:**

Determinar, por meio de análise biomecânica, os limites de corte patelar seguros para a reconstrução do ligamento cruzado anterior (LCA) e que minimizem riscos de fratura.

**Métodos:**

A partir de reconstrução tridimensional, foram feitos cortes triangulares na patela, com profundidade de 6,5 mm e largura e comprimento variáveis (8 a 12 mm e 10 a 20 mm), respectivamente, com intervalo de 1 mm). As combinações dos cortes constituíram 55 modelos para ensaios, com 5 variações de largura e 11 variações de comprimento, ensaiados por meio do método dos elementos finitos (MEF).

**Resultados:**

A média dos valores da máxima principal localizada (força de tração) foi de 4,36 Pa (DP 0,87 ± 0,76), e a mínima principal localizada (força de compressão) foi de −4,33 Pa (DP 1,05 ± 1,11). Comparando largura e comprimento à força de tensões dos valores da máxima principal, houve significância estatística a partir de 11 mm para largura e 13 mm para comprimento.

**Conclusão:**

Na reconstrução do LCA, a retirada do fragmento ósseo patelar mostrou-se segura para fragmentos menores que 11 mm de largura e 13 mm de comprimento, o que corresponde a 24% da largura e 28% do comprimento da patela utilizada. **Nível de Evidência II, Estudo Prospectivo Comparativo.**

## INTRODUCTION

Among many techniques for the reconstruction of the anterior cruciate ligament (ACL), bone-patellar tendon-bone autograft remains commonly used, ^(^ ^1^ only behind hamstrings autografts. ^(^ ^2^ ^), (^ ^3^ The main advantages of autograft techniques are the easy reproducibility, graft resistance, and the fixation and consolidation between host bone and bone block of the graft. ^(^ ^4^ Some negatives of the technique are postoperative pain in the anterior knee, difficulty of kneeling, and possible fracture of the patella and rupture of the patellar tendon. ^(^ ^1^ ^), (^ ^5^

ACL reconstruction results are very positive since normal function is restored in 90% of patients, enabling the return to sports activities in up to 80% of cases. However, many complications may arise with the procedure, regardless of the technique used, such as anterior knee pain, joint stiffness, secondary meniscal injury, pain around the graft fixation point, graft rupture, and patella fracture, which is rare but the most frequent when using bone/patellar/bone graft. ^(^ ^6^ ^), (^ ^7^

Some proximal bone block shapes reduce the risk of fracture, such as triangular, trapezoid, cylindrical and rectangular. ^(^ ^8^ Studies show that the shape of the block is unrelated to patellar fracture as long as the graft removal techniques are respected, that is, not exceeding 25 to 30 mm in length, 9 to 12 mm in width or one third of the tendon width, and 6 mm depth or one third of the patella depth. ^(^ ^9^ ^), (^ ^10^ Moreover, even after using the appropriate technique, patella resistance reduction ranges from 30 to 40%,^9^ ^), (^ ^11^ because the patellar dimensions are very variable depending on the patient’s gender, height and ethnicity. Establishing a fixed graft size pattern can be a variable to make the patella susceptible to fracture.

This study aimed to determine the safe patellar cut-off limits in ACL reconstruction that minimize fracture risks by biomechanical analysis using the whole finite element method (FEM) with several dimensions, demonstrating the results obtained in absolute value and percentile. 

## METHODS

Tomographic images of a left patella, 45 mm wide, 43 mm long, and 20 mm thick, in its greatest measurements, were used in this study. The images were extracted from the synthetic model 1145-70 of large size, from the Sawbone brand, composed of cortical and spongy bones manufactured in polyurethane, which were filed in the communication protocol that encompasses Digital Imaging and Communications in Medicine (DICOM) and used an Emotion tomography (16 channels, Siemens^™^, Munich, Germany) with 512 × 512 resolution and 1.0 mm between cuts. The file was imported into the InVesalius^™^ program for three-dimensional (3D) reconstruction of the anatomical structure. The program generated 3D files in STereoLithography (STL) format, sometimes also referred to as Standard Triangle Language (STL). 

The 3D virtual models of each system (bone and ligament) were started using the Rhinoceros^™^ 6 program (Robert McNeel & Associates, United States) and the MEF was performed in the SimLab™ program (HyperWorks, United States) using the Optistruct solver.

**Figure 1:**
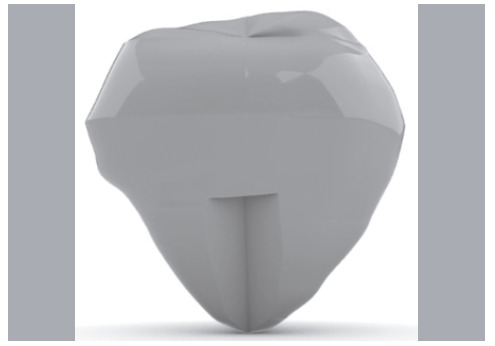
Frontal view of the patella, which shows the graft triangle in detail.

Triangular-shape cuts were made in the patella to simplify the technique applied-clinically, rectangular and trapezoidal shapes are more commonly used-, always with the same 6.5 mm depth. This shape is the one that most resembles the graft in a narrow-base trapezoidal form, which is usually obtained during the extraction of the graft during surgery. The width and length were the variables for this study, generating a gap that simulated graft removal, usually used in ACL reconstruction, with the ligament (Figure 1). The variation in length of the cuts was from 10 to 20 mm and in width from 8 to 12 mm, both with a 1 mm interval. Cut combinations constituted 55 models for testing, 5 variations of width and 11 of length.

For the simulations, after the removal of the gap that simulates the patellar graft, all models were imported into the Simlab^™^ program to run the test by the FEM. First, the individual identification of each part of the digital models (cortical bone, spongy bone, and ligament) was performed. The meshes were then controlled by each part, always maintaining the size of the element, to avoid contact problems between the different parts in the simulations. The element adopted for the formation of the meshes was the tetrahedral and the number of nodes and elements was also defined.

Knowing and defining the modulus of elasticity and Poisson’s coefficient of the materials of each part of the digital models was required for the simulations, as follows: cortical bone 17,000 MPa and 0.26 v; trabecular bone 1,700 MPa and 0.26 v; and ligament 1,200 MPa and 0.45 v, respectively.

**Figure 2:**
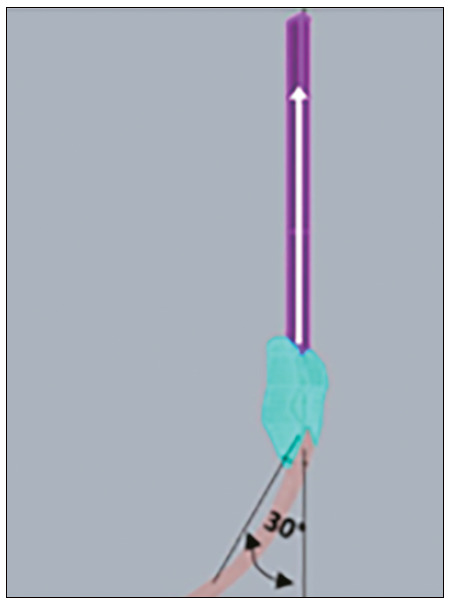
Vector-illustration of the traction force applied to the quadricipital tendon, cranial direction, with distal fixation, and inclination of the patellar tendon of 30°.

**Figure 3:**
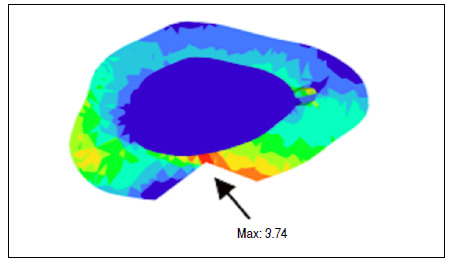
Tension region at the removal point of the graft.

The tests were performed by traction force in the quadricipital tendon, in the cranial direction, with distal fixation, and 30° inclination of the patellar tendon, which exerted a force on the patella-simulating a flexed knee and tensioning the anterior face of the patella and compressing the posterior face. From these conditions, the values of the total main maximum (traction force) and main minimum (compression force) were obtained and located on the graft gap for each combination (Figures 2 and 3).

**Figure 4:**
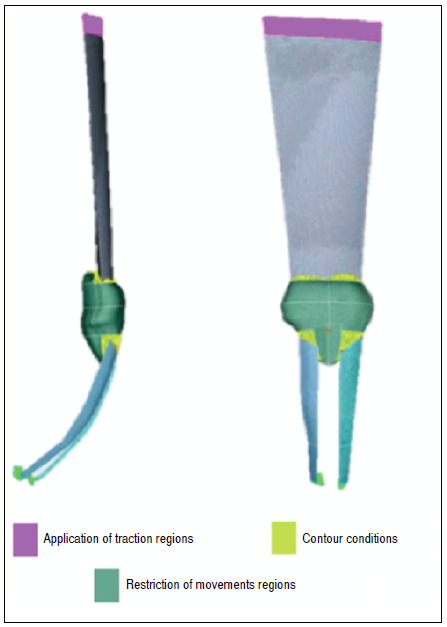
Contour conditions and load application regions.

To define the contour conditions, 3,000 N traction loads were applied on the Y axis of the application regions. No loads were applied to the X and Z axes, only in the Y axis. Subsequently, the (fixed) motion constraint regions were delimited, marked in all directions of the X, Y, and Z axes (universal coordinates) of displacement and rotation. These restrictions ensure that the alignment of the system is perfect, without displacement and/or rotation (Figure 4).

The tension results were analyzed in an exploratory way, by central position and dispersion measures. The statistical analysis was performed by analysis of variance (ANOVA) to verify the effect of length and width regarding stresses. Tukey’s post-test was applied to compare the width and length compared to the tensile and tensile force. The analyses were implemented in the SAS program version 9.4. Significance was achieved when p < 0.05. The 55 models were tested respecting the same conditions and contours and the application loading.

## RESULTS

**Figure 5:**
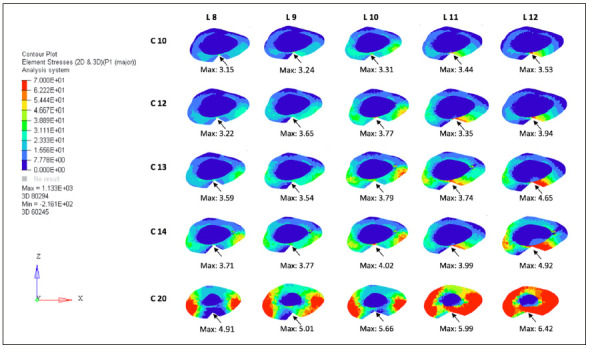
Association of patellar axial sections and their maxims located at the graft removal point.

The mean obtained from nodes and elements in the models was 296,606 and 183,719, respectively. The mean values of the localized maximum (traction force) were 4.36 Pa (SD 0.87 ± 0.76) and the minimum localized primary (compression force) was -4.33 Pa (SD 1.05 ± 1.11). Table 1 shows values of maximum and minimum for width and Table 2 shows these values for length. In Tukey’s post-test analysis comparing width and length regarding the tensile force of the main maximum values, statistical significance was found for graft widths starting from 11 mm, corresponding to 24% of the total width of the patella, regarding length, from 13 mm, 28% of the total patella length (Figure 5). Regarding the main minimum, no statistical difference was found for the values obtained, using One-way ANOVA (5%).

**Table 1:** Measurements of central position and dispersion of the maximum and minimum variables regarding width.

Variable	Width	N. Obs.	Mean	Stand. Dev.	Median	Q1	Q3	Minimum	Maximum	P-value
Max	8	11	3.95	0.63	4.01	3.22	4.51	3.15	4.91	0.0089
	9	11	4.05	0.6	3.94	3.54	4.68	3.24	5.01	
	10	11	4.33	0.74	4.35	3.77	4.87	3.31	5.66	
	11	11	4.37	0.86	4.41	3.5	4.98	3.35	5.99	
	12	11	5.14	1.06	5.2	3.94	6.09	3.53	6.42	
Min	8	11	- 3.89	1.09	- 4.15	- 4.8	- 3.02	- 5.2	- 1.95	0.3852
	9	11	- 4.47	0.83	- 4.54	- 5.12	- 4.01	- 5.7	- 2.98	
	10	11	- 4.52	0.87	- 4.65	- 5.3	- 3.87	- 5.69	- 2.99	
	11	11	- 4.59	0.97	- 4.48	- 5.48	- 4.07	- 6.12	- 2.98	
	12	11	- 4.58	0.97	- 4.54	- 5.48	- 4.01	- 6.15	- 2.99	

N. Obs.: number of samples observed; Stand. Pad.: standard deviation; Q1: first quartile; Q3: third quartile.

P-value for ANOVA.

**Table 2:** Measurements of central position and dispersion of the maximum and minimum variables regarding width.

Variable	Length	N. Obs.	Mean	Stand. Dev.	Median	Q1	Q3	Minimum	Maximum	P-value
Max	10	5	3.33	0.15	3.31	3.24	3.44	3.15	3.53	< 0.001
	11	5	3.44	0.22	3.4	3.33	3.5	3.18	3.78	
	12	5	3.59	0.3	3.65	3.35	3.77	3.22	3.94	
	13	5	3.86	0.45	3.74	3.59	3.79	3.54	4.65	
	14	5	4.08	0.49	3.99	3.77	4.02	3.71	4.92	
	15	5	4.38	0.5	4.35	4.01	4.41	3.94	5.2	
	16	5	4.64	0.67	4.49	4.21	4.57	4.13	5.78	
	17	5	4.84	0.63	4.69	4.45	4.78	4.37	5.92	
	18	5	5.03	0.62	4.87	4.68	4.98	4.51	6.09	
	19	5	5.25	0.67	5.15	4.7	5.4	4.69	6.33	
	20	5	5.6	0.64	5.66	5.01	5.99	4.91	6.42	
Min	10	5	- 5.77	0.39	- 5.7	- 6.12	- 5.69	- 6.15	- 5.2	< 0.001
	11	5	- 5.42	0.17	- 5.48	- 5.51	- 5.45	- 5.56	- 5.12	
	12	5	- 5.26	0.32	- 5.3	- 5.48	- 5.12	- 5.61	- 4.8	
	13	5	- 4.88	0.31	- 5.01	- 5.06	- 4.87	- 5.09	- 4.35	
	14	5	- 4.71	0.32	- 4.85	- 4.87	- 4.77	- 4.9	- 4.15	
	15	5	- 4.57	0.08	- 4.54	- 4.65	- 4.54	- 4.65	- 4.48	
	16	5	- 4.21	0.2	- 4.23	- 4.34	- 4.21	- 4.4	- 3.88	
	17	5	- 3.93	0.45	- 4.06	- 4.12	- 4.06	- 4.25	- 3.14	
	18	5	- 3.8	0.44	- 4.01	- 4.01	- 3.87	- 4.07	- 3.02	
	19	5	- 3.17	0.4	- 3.25	- 3.41	- 3.2	- 3.51	- 2.5	
	20	5	- 2.78	0.46	- 2.98	- 2.99	- 2.98	- 2.99	- 1.95	

N. Obs.: number of samples observed; Stand. Pad.: standard deviation; Q1: first quartile; Q3: third quartile.

P-value for ANOVA.

## DISCUSSION

The dissipation of internal stresses to the patellar body and the tension located in the focus of the gap of the graft removal followed an inverse pattern compared to the total stresses observed in the test body. The larger the graft, the more the total stresses decreased, due to the reduction of the total mass of the studied structure. However, the same did not occur when the localized stresses were observed.

The 6.5 mm depth for the graft was defined because it represented the mean between 6 and 7 mm presented as safety values in the literature. Values below 6 mm were excluded since they could not be applied from the clinical point of view, or above 7 mm because it is beyond 30% of the patellar thickness dimension applied in this study. ^(^ ^7^ ^), (^ ^9^

The variation of the cuts in length (10 to 20 mm) differ from that described in the literature as a safe dimension for graft removal, which is from 25 to 30 mm in length. ^(^ ^9^ This fact reflects the need of further studies, since we found a statistical significance from 13 mm, or 28%, in the results obtained, and may observe patellae of larger dimensions in studies that described dimensions from 25 to 30 mm as safe, confirming that demonstrating values in percentiles is essential. The variation in width (8 to 12 mm) adopted by the authors is similar to that described in the literature (9 to 12 mm). ^(^ ^7^ ^), (^ ^9^ Other factors that denote the importance of the observations of the safety percentiles described in this study is the optimization of the surgical objectives with the size of the grafts removed, the optimization of the contact of the graft with the tunnel to be performed and the safety of that of noble structures surrounding the donor site. ^(^ ^12^

At 15% of the gait cycle, a peak of quadriceps strength and knee angulation in flexion from 20 to 30° occurred, which in previous descriptions in the literature corresponds to 1.5 to 2 times body weight. ^(^ ^13^ ^), (^ ^14^ The 30° flexion positioning adopted by the authors, between the center of the patella and the fixation of the patellar tendon, corroborates the possibility of a higher incidence of burden on the patella. The 3,000 N traction load in the quadricipital tendon corresponds to three times the body weight of a 100 kg patient, being a local supraphysiological load application. Although the patellar fracture in the reconstruction of the ACL most commonly occurs during its removal, the authors aimed to present a safety limit of the graft size and its possible post operative weaknesses. ^(^ ^11^ ^)^ Thus, such an assessment of positioning and loads was adopted.

The limitations of this study consist of excluding the presence of cartilage on the surfaces, in the anatomical differences of contacts of the different types of trochlear surface, in the absence of mechanical properties of the synovial fluid, and in the absence of ligament actions and meniscal structure, which could be mitigating factors to the stress forces studied. Moreover, the possibility of the presence of notches in the angles of the cuts was not considered, a common occurrence in the use of vibratory saws in the removal of the patellar graft.^8^

The results are not intended as conduct determinants products. However, values of graft removal length with statistical significance (13 mm in length mentioned above) and significantly lower than those presented by the current literature and safety (25-30 mm)^10^ highlight the need to evaluate, both by prospective clinical studies and by review evaluations of patients who suffered patellar fracture after ACL reconstruction with patellar graft, the real safety predictive factors related to the dimensions of patellar grafts in ACL reconstruction.

## CONCLUSION

In this in vitro analysis, the removal of the patellar bone fragment from the ACL reconstruction proved to be safe in fragments smaller than 11 mm in width and 13 mm in length (maintaining a constant 6.5 mm depth), which corresponds to 24% of the width and 28% of the length of the patella used.
